# Reply to: High levels of plasma biomarkers at 24 h were found to be strong predictors of 90-day mortality: beware of some potential confounders!

**DOI:** 10.1186/s13613-021-00839-z

**Published:** 2021-03-15

**Authors:** Toni Jäntti, Veli-Pekka Harjola, Mikko Haapio, Johan Lassus

**Affiliations:** 1 Department of Cardiology, Heart and Lung Center, University of Helsinki, Helsinki University Hospital, HUS, 00029 Helsinki, Finland; 2 Department of Emergency Medicine and Services, University of Helsinki, Helsinki University Hospital, Helsinki, Finland; 3 Department of Nephrology, Abdominal Center, University of Helsinki, Helsinki University Hospital, Helsinki, Finland

To the Editor,

We wish to thank Honore et al. [1] for their interest in our article [2] and raising a caveat concerning the possible effect of renal replacement therapy (RRT) on proenkephalin (P-PENK) and neutrophil gelatinase-associated lipocalin (P-NGAL) levels in plasma. As suggested by Honore et al., this confounding could lead to an underestimation of the association of early P-PENK and P-NGAL levels with mortality so that if true, the association described in our article could in reality be even stronger. However, as the removal of solutes by RRT is affected also by factors other than molecular weight (such as charge, albumin binding capability, the type of membrane and RRT technique used) whether a molecule is removed from plasma by RRT and to which extent is difficult to predict and should be empirically tested. One small study performed in septic patients with AKI did not detect NGAL (or KIM-1) in the dialysate of continuous RRT [3], but data on the effect of RRT on plasma levels of the biomarkers in our study are mostly lacking.

In our study the time of onset of RRT was recorded in 17/22 patients and only 7 patients (5% of all patients included in our study) had RRT performed within 24 h of study baseline. Considering the small number of patients in the study who had RRT within 24 h of baseline, we believe that this would not affect the overall results.

Specifically for patients undergoing continuous RRT, using P-PENK, P-NGAL or any other biomarker that could be removed from circulation by RRT as a risk marker might lead to an underestimation of risk and should be interpreted with caution. We completely agree with Honore et al. that studies assessing the performance of prognostic biomarkers in this specific patient group are lacking and should be targeted for future studies.

## Data Availability

Not applicable.

## Abbreviations

P-PENK: Plasma proenkephalin
P-NGAL: Plasma neutrophil gelatinase-associated lipocalin
RRT: Renal replacement therapy
